# Pancreatitis and Acute Liver Failure From Coricidin® HBP Intoxication

**DOI:** 10.7759/cureus.10202

**Published:** 2020-09-02

**Authors:** Marsha Medows, Catalina Acosta, Vivian Vega

**Affiliations:** 1 Pediatrics, Woodhull Medical Center, Brooklyn, USA; 2 Pediatrics, New York University School of Medicine, New York, USA

**Keywords:** toxicology, liver failure, overdose, inpatient pediatrics

## Abstract

The use of over-the-counter medications as recreational drugs of abuse in adolescents is increasing. We present the case of a patient who presented with abdominal pain after the ingestion of Coricidin®, an over-the-counter cold medication that contains acetaminophen, chlorpheniramine maleate, and dextromethorphan hydrobromide. The case was complicated by acute liver failure and concomitant pancreatitis that, in a few reported cases, has been associated with high doses of acetaminophen.

## Introduction

During the last decade, there has been a surge in the recreational abuse of over-the-counter (OTC) cold medications, such as dextromethorphan, in adolescents [1]. These medications are used as recreational drugs at high doses due to its euphoric and hallucinogen properties similar to those of lysergic acid diethylamide [2]. The most commonly used product is Coricidin® HBP. which has been reported in approximately 87% of the abuse dextromethorphan cases [3,4]. Each tablet of Coricidin contains acetaminophen 350 mg, chlorpheniramine maleate 2 mg, and dextromethorphan hydrobromide 10 mg. This case report illustrates a patient with acute liver failure (ALF) and concomitant pancreatitis in the setting of utilizing Coricidin for recreational purposes. 

Acute pancreatitis is a serious condition, where there is a development of inflammation and necrosis of the parenchymal and peripancreatic tissue. This condition can be life threatening and has been linked to multiple etiologies, including gallstones, infections, trauma, autoimmune conditions, alcohol use, and drug consumption [5]. More than 130 medications have been associated with drug-induced pancreatitis [6]. In previous studies, people at the extremes of life and female sex have been identified as risk factors for developing drug-induced pancreatitis [7,8].

In children and adolescents, acetaminophen is the most common cause of medication-related ALF in the United States [9]. ALF is defined differently than in the adult population and is currently defined by the following criteria: (a) no evidence of prior liver disease; (b) coagulopathy that is not corrected by parenteral administration of vitamin K; (c) hepatic encephalopathy if the uncorrected prothrombin time (PT) or an international normalized ratio (INR) is between 15 and 19.9 seconds or 1.5 to 1.9, respectively; (d) hepatic encephalopathy was not required if the PT was ≥ than 2 seconds or INR was ≥2.

## Case presentation

We present a 15-year-old female patient with a past medical history pertinent for major depressive disorder and anxiety disorder, who was brought to the emergency department by emergency medical services with a primary concern of abdominal pain. The pain began approximately two hours before her presentation, and she described it as diffuse and stabbing with a 10/10 severity on the visual analog pain scale. She also indicated the pain was nonradiating, not exacerbated with movements, and associated with nausea and multiple episodes of nonbilious, nonbloody emesis. The patient did not report any history of diabetes, pancreatitis, gallbladder stones, trauma, or autoimmune disorders. At the time, the patient did not report the use of any medications.

The patient was alert and cooperative. Her initial vital signs were within reference ranges except for slightly elevated blood pressure (128/86 mmHg) and respiration rate (21 breaths per minute). We noted dry oral mucosa, but no other findings of the physical examination were remarkable. Her initial blood work was remarkable for a minor elevation in aspartate transaminase (AST)/alanine transaminase (ALT) and a positive acetaminophen level (Table 1).

**Table 1:** Laboratory values Na = sodium, K = potassium, Cl = chloride, CO_2_ = carbon dioxide, BUN = blood urea nitrogen, Ca = calcium, ALP = alkaline phosphatase, AST = aspartate amino transferase, ALT = alanine transaminase, THC = tetrahydrocannabinol, WBC = white blood cell count, Hb = hemoglobin Hct = hematocrit, MCV = mean corpuscular volume, Plt = platelet, N = neutrophil, L= lymphocyte, TSH = thyroid-stimulating hormone, T4 = total tyroxine, PT = prothrombin time, PTT = partial thromboplastin time, INR = international normalized ratio, PCO_2_ = partial pressure of carbon dioxide, PO_2_ = partial pressure of oxygen, HCO_3_ = bicarbonate, BE = base excess, O_2_ saturation = oxygen saturation, ND = no data

		Hospital Day 1	Hospital Day 2		Hospital Day 3	
Analyte		5:45 AM	10 AM	11 PM	3 AM	6 AM
Basic metabolic panel	Na (mmol/L)	136	-	-	138	-
	K (mmol/L)	3.7	---	-	3.2	-
	Cl (mmol/L)	99	-	-	103	-
	CO_2 _(mmol/L)	20	-	-	19	-
	Glucose (mg/dL)	130	-	-	100	-
	BUN (mg/dL)	9	-	-	2	-
	Creatinine (mg/dL)	0.7	-	-	0.52	-
	Ca (mg/dL)	10.3	-	-	8.7	-
	Anion gap (mEq/L)	17	-	-	16	-
Salicylate (mg/dL)		<0.3	-	-	-	-
Acetaminophen (µg/mL)		42.6	<5	-	<5	-
Ethanol (mg/dL)		<10	-	-	-	-
Liver function test	Albumin (g/dL)	4.9	4.4	-	4.2	-
	Total protein (g/dL)	7.6	6.7	-	6.1	-
	Total bilirubin (mg/dL)	1.7	3.5	-	2.6	-
	Direct bilirubin (mg/dL)	0.3	0.5	-	0.5	-
	ALP (U/L)	89	84	-	75	-
	ALT (U/L)	67	460	-	6,758	-
	AST (U/L)	71	449	-	7,997	-
	Globulin (g/dL)	2.7	2.3	-	1.9	-
	Alb/Glob (ratio)	1.8	1.9	-	2.2	-
Urine drug panel	Barbiturates	Negative	-	-	-	-
	Benzodiazepine	Negative	-	-	-	-
	Cocaine	Negative	-	-	-	-
	Methadone	Negative	-	-	-	-
	Opiates	Negative	-	-	-	-
	THC	Negative	-	-	-	-
	Phencyclidine	Negative	-	-	-	-
	Methaqualone	Negative	-	-	-	-
	Propoxyphene	Negative	-	-	-	-
	Amphetamines	Negative	-	-	-	-
Point-of-care glucose (mg/dL)		122	-	-	-	-
Point-of care pregnancy		Negative	-	-	-	-
Complete blood count	WBC (mcl)	7.72	-	10.51	-	-
	Hb (g/dL)	15.1	-	13.4	-	-
	Hct (%)	43.1	-	38.2	-	-
	MCV (fL)	85.3	-	83.6	-	-
	PLT (mcl)	425	-	268	-	-
	N (%)	70.3	-	92.9	-	-
	L (%)	19.3	-	2.5	-	-
Thyroid function tests	TSH (uIU/ml)	ND	-2.08	-	-	-
	Total T4 (ug/dL)	ND	10.1	-	-	-
ECG		Sinus bradycardia, otherwise, within reference ranges	-	-	-	-
Coagulation tests	PT (seconds)	ND	21.4	-	27.6	-
	PTT (seconds)	ND	37.9	-	33.4	-
	INR (ratio)	ND	1.89	-	2.42	-
Lipase		ND	-	>600	-	-
Venous blood gas	pH	ND	-	-	-	7.39
	PCO_2 _(mmHg)	ND	-	-	-	43
	PO_2 _(mmol/L)	ND	-	-	-	25
	HCO_3_ (mmol/L)	ND	-	-	-	25
	BE (mmol/L)	ND	-	-	-	0.1
	O_2 _saturation (%)	ND	-	-	-	45

Two hours after the patient’s arrival at the hospital and upon further questioning, she disclosed that the night before her presentation, she ingested 20 pills of Coricidin. Additionally, she reported that she ingested the pills with an energy drink for recreational purposes and denied any other recent consumption of drugs or alcohol. The Center for Poison Control was contacted, and treatment with N-acetylcysteine (NAC) was started. She received NAC at an initial loading dose of 150 mg/kg over one hour, followed by a second dose of 50 mg/kg over four hours.

Five hours after starting the NAC regimen, a second laboratory assessment showed an increase in AST (460 U/L) and ALT (449 U/L; Table 1). She continued to receive a third and fourth dose of NAC at 100 mg/kg, infused over 16 hours each.

On day 2 of hospitalization, the patient developed intermittent abdominal pain, this time located in the epigastric region. She described the pain as stabbing, nonradiating, with an intensity of 10/10 and exacerbated with movements. Her physical examination revealed active bowel sounds, no abdominal distention, and was notable for epigastric tenderness with superficial palpation with no rebound or guarding. We noted no jaundice, and she had a preserved mental status. Additional blood work revealed elevated lipase (>600 U/L). Routine management for pancreatitis was started with intravenous fluids. Biochemical studies obtained two hours before finalizing the fourth NAC infusion are detailed in Table 1.

On hospital day 3, the patient’s blood work was significant for a marked increase in AST/ALT levels. We contacted the Center for Poison Control again, and they suggested contacting the hepatic transplant team for transfer and further management of the patient. Finally, the patient was transferred to an external institution for evaluation by a liver transplant team. Based on the patient’s notes from the outside hospital team, the patient was admitted for observation, given supportive treatment, and had a gradual decrease in liver enzymes and lipase levels over the next two days. The patient did not require liver transplantation and was discharged on hospital day 6.

## Discussion

Drug-induced pancreatitis cases account for a small percentage of acute pancreatitis cases in general, with a reported incidence of 0.1% to 2% [1,6]. Despite the low incidence rate, drug-induced pancreatitis remains a serious condition with an estimated mortality rate of 30% [5]. Recreational use of OTC cough syrups and tablets containing dextromethorphan is an emerging drug abuse trend among teenagers [9]. These medications are consumed at high doses to achieve hallucinogenic effects. Several adverse effects have been observed from these high doses, including nausea, vomiting, ataxia, hallucinations, seizures, and respiratory depression [10,11].

Other pharmacological ingredients common to OTC cold and cough products, such as chlorpheniramine maleate and acetaminophen, can have adverse effects. Chlorpheniramine maleate has anticholinergic effects, such as urinary retention, dry mouth, tachycardia, and confusion [12]. Acetaminophen is well known to cause hepatic toxicity at high doses, as seen in our case; however, cases of acetaminophen-induced pancreatitis are extremely rare. Acetaminophen needs to be recognized as a potential etiologic agent after other causes of pancreatitis have been ruled out. The mechanism of acetaminophen-induced pancreatitis remains unknown [6]. Neither dextromethorphan nor chlorpheniramine maleate has been linked to drug-induced pancreatitis.

## Conclusions

This case highlights the need for physicians to be aware of the increasing use of OTC cough syrup and tablets as an abused drug, especially in young adolescents. The treatment for acute intoxication with these medications is usually supportive care. The recognition of potential complications from the other components of these preparations, like acetaminophen, is crucial for optimal patient outcomes.
